# Monoterpenoids Evolution and MEP Pathway Gene Expression Profiles in Seven Table Grape Varieties

**DOI:** 10.3390/plants11162143

**Published:** 2022-08-18

**Authors:** Xiaomiao Zhou, Songyu Liu, Wengping Gao, Binfang Hu, Baoqing Zhu, Lei Sun

**Affiliations:** 1 Beijing Advanced Innovation Center for Tree Breeding by Molecular Design, Beijing Forestry University, Beijing 100083, China; 2 Department of Food Science and Engineering, College of Biological Sciences and Biotechnology, Beijing Forestry University, Beijing 100083, China; 3 Institute of Forestry and Pomology, Beijing Academy of Agricultural and Forestry Sciences, Beijing 100093, China

**Keywords:** table grapes, monoterpenoids, GC-MS, *VvDXS* expression, MEP pathway

## Abstract

This research investigated the evolution of both monoterpenoids and expression profiles of related biosynthesis genes in the MEP pathway in seven different table grape varieties from veraison to maturity stage in two seasons, and the correlation was further evaluated between monoterpenoid accumulation and expression of these genes studied in these varieties. Results showed that linalool, *trans*-furan linalool oxide, geraniol, and *cis*-furan linalool oxide were the main compounds in the five Muscat varieties two seasons. ‘Zaomeiguixiang’ had the highest contents of geraniol and *β*-Citronellol. ‘Xiangfei’ had the most abundant of linalool and *cis*-furan linalool oxide, whereas the neutral varieties of ‘Moldova’ and ‘Christmas Rose’ had the least amount. Monoterpenoid volatiles have been grouped in three evolutionary patterns in the berry development of these varieties. ‘Zaomeiguixiang’ and ‘Xiangfei’ had distinct different pattern of terpenoids evolution profiles. Pearson’s correlation analysis showed that in the MEP pathway, the first biosynthesis gene *VvDXS3* was significantly correlated to the accumulation of monoterpenoids, and appeared to be an important candidate gene for synthesis of the monoterpenoids.

## 1. Introduction

The grape is a widely cropped variety with 77.8 million tons of global output, of which the percentage of table grapes is 36%, according to the OIV (Organisation Internationale de la Vigne et du Vin, 2022). China is one of the most important table grape markets in the globe and table grapes account for about 80% of China’s total grape production [1]. Consumers in China like the table grapes with red to pink color and sweetness mouthfeel. Berries with specific flavor are becoming more and more popular in the market in recent years [2]. Aroma is the important sensory parameter for grapes. There were kinds of aroma compounds found in grape berries including terpenoids, norisoprenoids, 2-methoxy-3-methylpyrazine, aldehydes, alcohols, and esters [3,4]. Some monoterpenoids could desorb a flora flavor, also called Muscat, which was easily felt by the human olfactory organ [5,6].

In grape berries, the monoterpenoids mainly exist in pericarp [7]. Their contents was determined by the genotype [8], and affected by development stage [9], environment and management [10,11,12]. There are two forms of monoterpenoids, free form and bound form. The former can contribute directly to the flavor, while the latter, which is a class of potential aroma compound, can be converted to free form by hydrolysis [13,14,15]. However, most previous studies focused on wine grapes but not on table grapes.

Biologically, the biosynthesis of geranyl diphosphate (GPP), the precursor terpenoids in plants, is mainly through two pathways, methyl-erythritol-4-phosphate pathway (DXP/MEP) in the plastid, and mevalonate pathway (MVA) in the cytoplasm [16]. It is generally believed that the GPPs for monoterpenoids biosynthesis in grape berries mainly come from the former pathway. The MEP pathway was found start from pyruvate and G3P (3-phosphate glyceraldehyde), by DXS (1-deoxy-D-xylulose-5-phosphate synthase), the first enzyme in the MEP pathway, and they are changed in to DXP (1-deoxy-D-xyulose-5-phosphate), then converted into GPP by six enzymatic reactions. Then, the GPP was synthesized in to monoterpenoids (C10) by terpene synthases (TPSs), mainly in plastid [17,18]. Previous studies showed that SNPs of *VvDXS1*, in particularly SNP at 1822 which led to K284N, could explain the difference of monoterpenoids contents among grape varieties to some extent [19,20].

In recent years, researchers have also found that the accumulation of monoterpenoids was consistent with the expression pattern of some genes in the MEP pathway during berry ripening. The related studies were also mainly focused on wine grapes. Laura Costantini et al. found that the monoterpenoids increased from veraison in ‘Moscato Bianco’ berries and it was consistent with the expression of *VvHDR* [21]. Ya-Qin Wen et al. had similar findings in ‘Muscat Blanc à Petits Grains’ [22]. Juri Battilana et al. found a positively correlated relationship between *VvDXS1* expression and monoterpenoids accumulation among ‘Moscato Bianco’ [19]. To our best knowledge, few studies were focused on table grapes. Wang et al. [23] found that in ‘Shine Muscat’, terpenoids gradually increased during fruit development while a relatively decline in abundance of total terpenoids was observed during maturity. *VvDXS1*, *VvDXS3*, *VvDXR*, and *VvHDR* were found to be positively correlated with terpenoid accumulation. JI Xiao-hao et al. [24] found that the monoterpenoids concentrations increased during berry ripening in two table grapes (‘Kyoho’ and ‘87-1’) two table grapes. Correlation analysis revealed a positive correlation between the expression of *VvDXS* and *VvDXR* with monoterpenoids content, while a negative correlation was found between the gene expression of *VvHDR* with monoterpenoids content. From above, these results indicated that although most of the monoterpenoids mainly accumulated during berry ripening in kinds of varieties, the expressional patterns of related genes in MEP pathway were found not the same.

The relationship between the monoterpenoids accumulation and expression patterns of different genes in the MEP pathway might be complex in varied table varieties. In this study, seven varieties of table grapes were selected, including ‘Christmas Rose’, ‘Muscat of Alexandria’, ‘Moldova’, ‘Italia’, ‘Zaomeiguixiang’, ‘Tamina’, and ‘Xiangfei’ in two consecutive seasons (2013 and 2014). These varieties were found with different sensory features. The gene expression in the MEP pathway was analyzed to explain the role of key genes of the MEP pathway on the accumulation of monoterpenoids in these table grape varieties. In each season, the composition of monoterpenoids was studied for each variety from veraison to full maturity through the use of SPME-GC/MS (solid-phase microextraction-gas chromatography/mass spectrometry) techniques. Furthermore, the related gene expression profiles in the MEP pathway were investigated by qPCR in 2014 season. This research could help us elucidate the contribution of the monoterpenoids to the Muscat flavor of table grapes, and further identify the key genes controlling the monoterpenoids accumulation in kinds of table grapes during the development of berries.

## 2. Results and Discussion

### 2.1. Monoterpenoids Content, Flavour Contribution, Variety and Season Effect

Monoterpenoids were found accumulated in the berries during the grape development stages, and their contents at fully maturity stage would determine the overall Muscat aroma of grape berries. Therefore, it is important to study their concentration at harvest point. In this study, there were 25 monoterpenoids found in these seven table grapes at harvest. (Table 1 and Table 2). According to their structures, these compounds included 1 cyclic acid: geranic acid; 5 cyclic alkenes: *β*-*trans*- ocimene, *β*-*cis*- ocimene, allo-ocimene, (*trans*,*cis*)-allo-ocimene, *β*-myrcene; 6 cyclic alcohols: linalool, isogeraniol, nerol, *β*-citronellol, geraniol, *γ*-geraniol; 3 cylic aldehydes: neral, citronellal, geranial; 6 ethers: *cis*-pyran linalool oxide, *trans*-rose oxide, nerol oxide, *cis*-furan linalool oxide, *cis*-rose oxide, *trans*-furan linalool oxide; 3 acyclic alkenes: *γ*-terpinen, limonene, terpinolene; 2 acyclic alcohols: *α*-terpineol, 4-terpineol. According to the research of Ruiz-Garcia [25], the existence of rose oxides was significantly related to the Muscat flavor, rose oxides could be detected in all the Muscat type varieties while not in neutral flavor varieties, the results of this study validated the above research. Meanwhile, our results were consistent with this study. Both *trans*-rose oxide and *cis*-rose oxide could be identified at harvest time (E-L38) in the five Muscat varieties of ‘Xiangfei’, ‘Tamina’, ‘Italia’, ‘Zaomeiguixiang’, and ‘Muscat of Alexandria’. It was worth noticing that only around 20 monoterpenoids were detected in ‘Moldova’ at ripening, and all the 25 monoterpenoids were detected in ‘Christmas Rose’; but four compounds including (*E*)-*β*-ocimene, *cis*-pyran linalool oxide, and especially *cis*-rose oxide and *trans*-rose oxide were in trace content and could not be quantified in these two neutral varieties.

Total monoterpenoid concentrations were highest in the ‘Xiangfei’ variety in both seasons of harvest (785.86 μg/L in 2013 and 1216.38 μg/L in 2014), then followed by the five variety of ‘Italia’, ‘Zaomeiguixiang’, ‘Christmas Rose’, ‘Tamina’, and ‘Muscat of Alexandria’, whereas the ripen ‘Moldova’ variety exhibited the least content (12.36 μg/L in 2013 and 0.75 μg/L in 2014). Regarding the alcohols, linalool and geraniol were the most abundant compounds while 4-terpineol, nerol and isogeraniol had the lowest concentration in both seasons; total alcohol concentration in 2014 was higher than in 2013. In terms of alkenes, all the eight detected alkenes were at very low level or not detected in ‘Moldova’ and ‘Christmas Rose’, and the concentration of them in the other five varieties ranged from 0.04–22.12 μg/L; ‘Italia’ and ‘Zaomeiguixiang’ had the similar content of alkenes in 2014. ‘Xiangfei’ had the most abundant of *trans*-furan linalool oxide and *cis*-furan linalool oxide in both seasons. For the two rose oxides, *trans*-rose oxide and *cis*-rose oxide were not detected in ‘Moldova’ and ‘Christmas Rose’, and even not abundant in the other five Muscat varieties, but they could contribute to the rose flavor due to the low odor threshold (0.5 μg/L). Regarding the 3 aldehydes, ‘Xiangfei’ had the most abundant of citronellal, while ‘Zaomeiguixiang’ had the most abundant of neral and geraniol; all the three aldehydes were not detected in the varieties of ‘Moldova’ and ‘Christmas Rose’ in 2014.

In this study, we carried out a two-way ANOVA to elucidate the effects of variety, season and variety × season interaction in ripening table grapes for monoterpenoids. (Table 3). The results showed that genotype plays a major influence in the composition for these compounds except for citronellal and *γ*-Terpinen, and that season also plays an important role in the content of most compounds except for (*trans*,*cis*)-allo-ocimene, *trans*-furan linalool oxide, *cis*-furan linalool oxide, citronellal, *β*-citronellol, nerol, and geraniol. Moreover, the Variety × Season interaction played a role in affecting the level of some of them. These results suggested that the seven table grape varieties showed significant differences not only in the total content of monoterpenoids but also in their composition.

### 2.2. Evolution of Monoterpenoids during Berry Development

**Figure 1:**
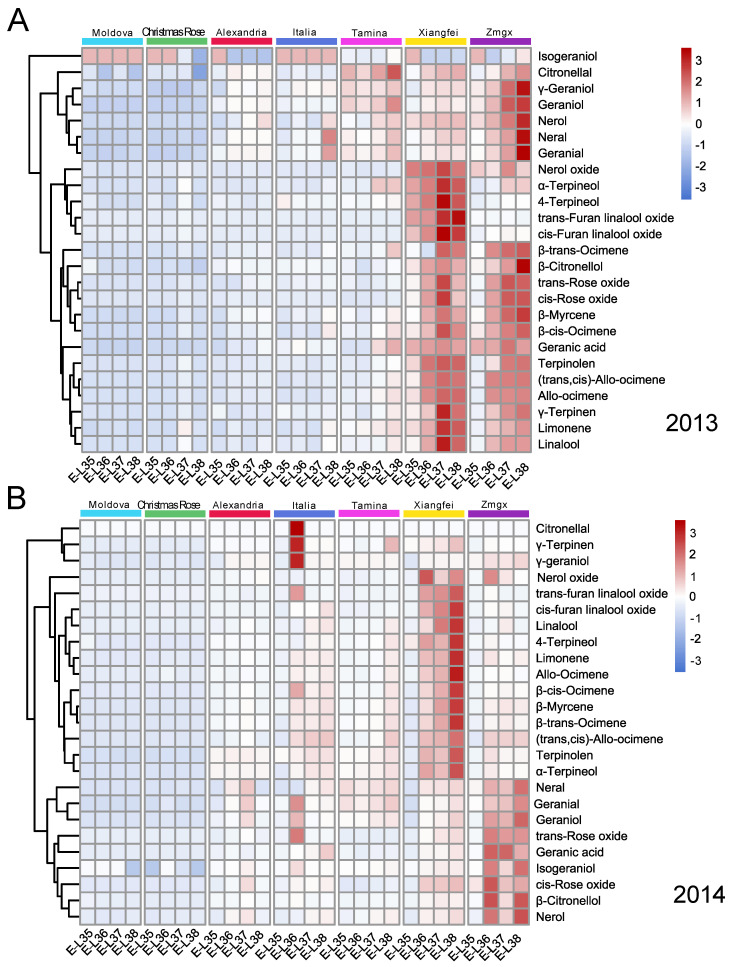
Heat plots of monoterpenoids of different table grape varieties during the development of berries in 2013 (**A**) and 2014 (**B**). ‘Alexandria’ represented ‘Muscat of Alexandria’, ‘Zmgx’ represented ‘Zaomeiguixiang’.

In 2013, for ‘Moldova’, *β*-*trans*-Ocimene, *β*-*cis*-Ocimene, *trans*-Rose oxide, and *cis*-Rose oxide were almost not detected during the berry development, most of the compounds decrease from veraison to maturity; ‘Christmas rose’ showed the similar evolution pattern (Figure 1A). And they exhibited the similar evolution pattern in the 2014 season (Figure 1B). These two varieties were categorized as neutral flavor, the C6 alcohol and aldehyde were their dominant compound according to our previous research, and the decrease trend of monoterpenoids was also consistent with the other neutral varieties of wine grapes, such as Shiraz and Cabernet Sauvignon [9].

For ‘Muscat of Alexandria’ and ‘Italia’, most of monoterpenoids accumulated from veraison to maturity, especially for the dominant compounds of Geraniol, *β*-Citronellol, *trans*-Furan linalool oxide, *cis*-Furan linalool oxide and linalool. ‘Tamina’ accumulated more content of neral, citronellol, *γ*-geraniol, and nerol. The content of geraniol, geranial, geranic acid, nerol, and neral in ‘Zaomeiguixiang’ were quite same or even higher than ‘Xiangfei’. Geranic acid showed a significant decrease near ripening in the above two varieties. Nerol oxide had similar accumulation pattern in ‘Italia’ and ‘Muscat of Alexandria’. The concentration of linalool increased during development except for the varieties of ‘Christmas Rose’ and ‘Moldova’; Linalool accumulated since veraison, then decreased slightly near maturity in ‘Xiangfei’ and ‘Muscat of Alexandria’. Linalool oxide was the cyclized form of linalool, and had four kinds of isomers including *trans*/*cis* and furan/pyran type, the content of linalool oxide in ‘Xiangfei’ were much higher than the other varieties, while *cis*-furan linalool oxide was not detected in all stages in ‘Moldova’ in the 2014 season. Geraniol might be transferred to citronellol by reduction reaction, then be cyclized to produce the rose oxide; Geraniol and Citronellol did not show any significant changes in these seven varieties except ‘Zaomeiguixiang’; *trans*-rose oxide and *cis*-rose oxide were not detected in the all stage in ‘Christmas Rose’ and ‘Moldova’, and remained at the low level in other five varieties.

These table grapes were clustered into three groups regarding the evolution similarity of monoterpenoids in both seasons (Figure 1). The two neutral grapes, ‘Christmas Rose’ and ‘Moldova’ belonged to type I, in which most of the monoterpenoids showed more or less decrease since veraison, but the total amount of monoterpenoids did not show any apparent fluctuation. ‘Tamina’, ‘Italia’ and ‘Muscat of Alexandria’ could be classified into type II, in which monoterpenoids increased since veraison, and the total concentration varied between 30 μg/L to 140 μg/L. Type III, including ‘Zaomeiguixiang’ and ‘Xiangfei’, showed a similar trend, and the total content of monoterpenoids ranged between 130 μg/L to 1200 μg/L. It was worth noticing that again ‘Zaomeiguixiang’ had higher content of citronellal, *γ*-geraniol, geraniol, neral, nerol, and geranial, while ‘Xiangfei’ had more abundant of neral oxide, *α*-terpineol, 4-terpineol, *trans*-furan linalool oxide, *cis*-furan linalool oxide, but for other compounds, these two varieties had similar trend during berry development.

**Figure 2:**
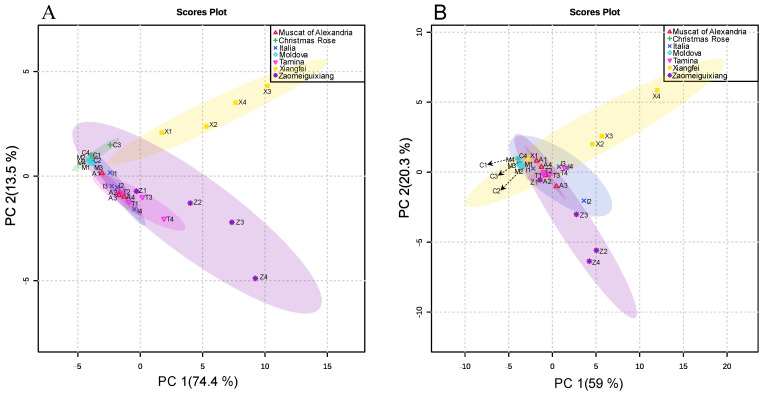
Principle component analysis of monoterpenoids composition in different table grape varieties during berry development in 2013 (**A**) and 2014 (**B**). A represented ‘Muscat of Alexandria’; C represented ‘Christmas Rose’; I represented ‘Italia’; M represented ‘Moldova’; T represented ‘Tamina’; X represented ‘Xiangfei’; Z represented ‘Zmeiguixiang’. 1 represented E-L35; 2 represented E-L36; 3 represented E-L37; 4 represented E-L38.

Principal component analysis (PCA) was carried out and the results were shown in Figure 2. The results suggested that the first component and the second component explained 74.4% and 13.5% of the total variance for 2013, and 59.0% and 20.3% for 2014 (Figure 2). The samples of E-L 36, 37 and 38 from ‘Xiangfei’ and ‘Zaomeiguixiang’ could be clearly separated from the rest samples. E-L 36, 37 and 38 of ‘Xiangfei’ samples were concentrated on the first quadrant and while E-L 36, 37 and 38 of ‘Zaomeiguixiang’ samples were located in the fourth quadrant (Figure 2A). Quite similar results were found in 2014 (Figure 2B).

### 2.3. Transcript Level of MEP Pathway Key Genes during Development

Biochemical studies showed that the monoterpenoids were mainly produced by the MEP pathway during berry development and it had been documented that DXS and DXR were found as the key enzymes that could regulate the biosynthesis of monoterpenoids in some table grapes [3,21,23]. In order to explain their roles on the accumulation of monoterpenoids in the seven table varieties used in this study, we analyzed their transcript levels except ‘Moldova’ and ‘Italia’ in 2014 season.

**Figure 3:**
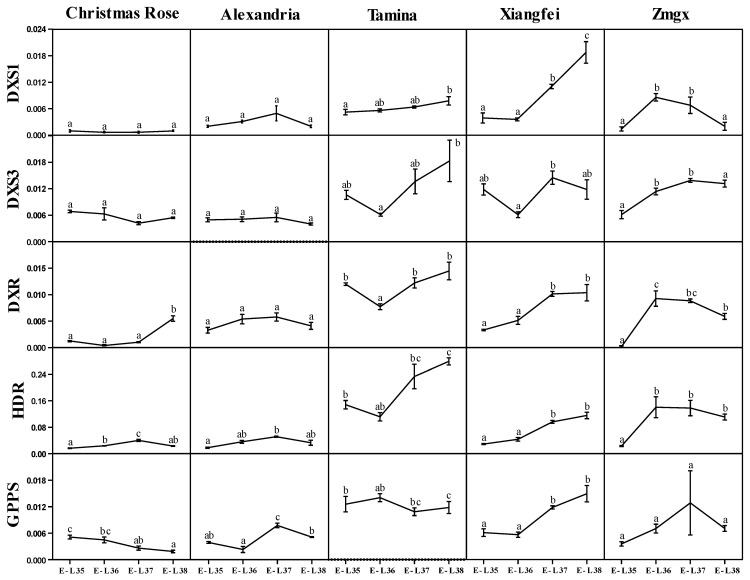
Transcript levels of critical genes of the MEP pathway during the development of berries in different table grape varieties in 2014. Values with different letters (a–c) on a row were significantly different based on Tukey’s multiple range test (*p* < 0.05). “Zmgx” represents “Zaomeiguixiang”, and “Alexandria” represents “Muscat of Alexandria”. Each gene expression level was expressed as a ratio relative to the E-L 35 stage of the “Xiangfei” variety (the ratio was set to 1).

It was reported that a single nucleotide polymorphism (SNP) mutation in *VvDXS1* gene could lead to the significant difference of monoterpenoids content in a specific grape variety [19]. In this study, increased expression of the *VvDXS1* during berry ripening was observed in the varieties of ‘Muscat of Alexandria’, ‘Tamina’, and ‘Xiangfei’. It was worthy to note that the expression of *VvDXS1* in ‘Xiangfei’ increased dramatically and reached at the highest level at maturity, consistent with some previous studies on table grapes, such as Xiaofeng Yue et al. (2021) in ‘Muscat Hamburg’ [26] and Wu Wang et al. (2020) in ‘Shine Muscat’ [27], while its expression in ‘Christmas Rose’ remained at a low level in the whole process. These results were consistent with monoterpenoids accumulation found in Section 2.2 (Figure 3). It was worthwhile to notice that *VvDXS1* showed a decreased expressional trend when approaching maturity, which was not well matched with monoterpenoids accumulation in ‘Zaomeiguixiang’ (Figure 3).

*VvDXS3* had low transcript levels in ‘Christmas Rose’ and ‘Alexandria’ varieties, while having higher expression in ‘Tamina’, ‘Xiangfei’, and ‘Zaomeiguixiang’. Wu Wang et al. [27] and Martin et al. [28] have found that *VvDXS3* was gradually up-regulated during berry development by qRT-PCR in ‘Sunshine Rose’ and ‘Gewurztraminer’, which were consistent with our findings in ‘Zaomeiguixiang’. The expression pattern of *VvDXS3* in the other four varieties was different from ‘Zaomeiguixiang’, with downregulation in ‘Christmas Rose’, no significant change in ‘Alexander’, a decrease and then an increase in ‘Tamina’, and a decrease and then an increase with a decrease again at maturity in ‘Xiangfei’.

*VvDXR* expression was increased during berry maturation in ‘Christmas Rose’, ‘Muscat of Alexandria’, and ‘Xiangfei’ from the veraison, which is consistent with previous findings in ‘Shine Muscat’ [23,27], ‘Moscato Bianco’ [21], and ‘Gewurztraminer’ [28]. Its expression was significantly higher in ‘Xiangfei’ than in the other two varieties, which is consistent with the pattern of monoterpenoids accumulation among these varieties found previously. The expression pattern in ‘Tamina’ decreased followed by an increase with the highest expression at maturity in line with the trend found in ‘Kyoho’ and ‘87-1‘ two table grapes [24]. The unique expression trend of *VvDXR* in ‘Zmeiguixiang’, rising at the beginning of veraison and gradually decreasing with berry maturity, was not consistent with the reported, but similar to its corresponding compounds accumulation pattern.

The expression pattern of *VvHDR* with progressively higher expression during berry ripening has been reported in ‘Shine Muscat’ [27], ‘Muscat Hamburg’ [29], ‘Moscato Bianco’ [21], ‘Muscat Blanc à Petits Grains’ [22], and ‘Gewurztraminer’ [28]. *VvHDR* gene expression was upregulated in ‘Kyoho’ during berry development, while ‘87-1’ had lower transcript levels slightly upregulated near maturity [24]. The expression pattern of *VvHDR* in ‘Xiangfei’ was consistent with the trend in ‘Kyoho’ and in ‘Christmas Rose’ and ‘Alexander’ was consistent with that in ‘87-1’. The expression patterns of decreasing at the veraison followed by gradual increase during berry ripening of *VvHDR* in ‘Tamina’ and up-regulation at the veraison followed by gradual decrease in ‘Zaomeiguixiang’ were not consistent with any previous reports.

The expression of *VvGPPS* in ‘Xiangfei’ was found gradually upregulated, consistent with in ‘Moscato Bianco’ [29] and ‘Muscat Blanc à Petits Grains’ [22]. Its expression in ‘Zaomeiguixiang’ and ‘Alexander’ increased with berry development and decreased near maturity and was similar to the trend reported in ‘Shine Muscat’ [23]. The expression of *VvGPPS* in ‘Christmas Rose’ was gradually downregulated and in ‘Tamina’ firstly increased and then decreased, different from other reports.

Based on the gene transcript levels, it could be found that *VvDXS3*, *VvDXR*, *VvHDR*, and *VvGPPS* were expressed at higher levels in’Tamina’, ‘Xiangfei’, and ‘Zaomeiguixiang’, while at lower levels in ‘Alexandria’ and ‘Christmas Rose’, the gene expression trends were consistent with monoterpenoid accumulation pattern among varieties found before.

### 2.4. Correlation of Genes Expression and Monoterpenoids Accumulation

To study the relation between the expression of genes in MEP pathway and the accumulation of monoterpenoids in all the seven varieties studied here, the Pearson’s correlation analysis was carried out (Table 4). It was found that the *VvDXS3* expression was strongly positively correlated with most of the 25 monoterpenoids except four of them, including *trans*-furan linalool oxide, *cis*-furan linalool oxide, nerol oxide, and 4-terpineol. This indicated that the differential expression of the *VvDXS3* might play an important role in diversifying the monoterpenoids profiles in table grape varieties. The high expression levels of *VvDXS1* and *VvDXS3* at the maturity stage might be responsible for the high concentrations of monoterpenoids in ‘Xiangfei’.

*VvDXR* and *VvGPPS* showed a positive correlation with all monoterpenoids; *VvHDR* had a positive correlation with 24 monoterpenoids but a negative correlation with *trans*-furan linalool oxide. In 2021 Wu Wang et al. [23] studied the correlation of the expression of genes in MEP pathway with monoterpenoids in ‘Shine Muscat’ and found that *VvDXR*, *VvGPPS*, and *VvHDR* were positively correlated with all detected monoterpenoids, which was contrary to our finding that *VvHDR* has a negative correlation with *trans*-furan linalool oxide, speculating that was due to the different varieties.

### 2.5. The Correlation of the Genotype snp1822 in VvDXS1 to the Composition of Monoterpenoids

According to the report of Emanuelli (2010), the gain of function mutation of *VvDXS* gene caused the accumulation difference of monoterpenoids in grape berry, the snp: 1822 G/T and T/T lead to the muscat phenotype while G/G leads to the neutral flavor [30]. This snp site in all the seven varieties studied here was genotyped by sanger sequencing (Figure S1). The results showed that ‘Christmas rose’ and ‘Moldova’ carried the G/G loci, which was corresponded to the very low content of total monoterpenoids, ‘Muscat of Alexandria’, ‘Italia’, ‘Tamina’ and ‘Xiangfei’ carried the G/T loci, while ‘Zaomeiguixiang’ carry the T/T loci. Emanuelli investigated the monoterpenoids contents of core collection in FEM to conclude that the homozygous mutants (T/T) had higher contents than heterozygous mutants (G/T) [30]. It was interesting to notice that ‘Xiangfei’ with G/T type had the highest total contents, more than ‘Zaomeiguixiang’ with T/T type.

The expressions of *VvDXS1* and *VvDXS3* were compared in the two varieties. It was worthy to notice that the expression of *VvDXS1* in the two varieties showed opposite trends during the developmental stage. In ‘Xiangfei’, it decreased and then increased sharply with a high expression at grape ripening, while in ‘Zaomeiguixiang’, it showed an increasing trend and then a continuous decrease with a very low expression in the ripening stage. The expression of *VvDXS3* was similar in both varieties, but had a slightly higher expression in ‘Xiangfei’at maturity. The expression of these *VvDXSs* might affect monoterpenoids accumulation within the two varieties.

## 3. Materials and Methods

### 3.1. Chemicals and Standards

The standards, including *β*-Myrcene (90.0%), Limonene (99.0%), terpinolene (94.0%), *cis*-rose oxide (96.0%), *cis*-furan linalool oxide (98.0%), linalool (96.0%), 4-terpinenol (95.0%), menthol (98.0%), *β*-citronellal (99.0%), myrtenol (95.0%), *α*-terpineol (99.0%), nerol (98.0%), neral, geraniol (97.0%), and geranial (98.0%), were obtained from Sigma-Aldrich (St. Louis, MO, USA). In total, 98% of 4-methyl-2-pentanol (internal standard) was also obtained from Sigma-Aldrich. The purified water used in this research was obtained from a Master Touch purification system (Shanghai, China). Polyvinylpolypyrrolidone (PVPP) was a product of huayueyang (Beijing, China). SYBR^®^ Premix Ex TaqTM and Plant Total RNA Kit were respectively purchased from TaKaRa Bio (Otsu, Shiga, Japan) and Gene-Better Life Science (Beijing, China). The reverse transcription system kit was purchased from Promega (Madison, WI, USA). All other reagents used in this study were obtained from the Beijing Chemical Works (Beijing, China), unless otherwise specified.

### 3.2. Sample Collection

Seven table grape varieties, including ‘Xiangfei’(VIVC:23248), ‘Moldova’(VIVC:7896), ‘Tamina’(VIVC:12244), ‘Italia’(VIVC:5582), ‘Zaomeiguixiang’(VIVC:24775), ‘Christmas Rose’(VIVC:2624), and ‘Muscat of Alexandria’(VIVC:8241), were grown at the experimental field in the Institute of Forestry and Pomology, Beijing Academy of Agricultural and Forestry Sciences in China (39°58′ N and 116°13′ E). Photographs of these varieties and their genealogical relationships are displayed in Figure S2. The self-rooted vines of these varieties were planted in the spring of 2008 by vertical shoot position trellis system with row and plant space of 2.5 m × 0.75 m. Simple rain shelter, ground cover horticultural ground cloth, drip irrigation water supply, and conventional pest and disease management patterns are used, mechanical burial overwintering. Pruning and fertilization management are consistent during the growing season. Regarding their pedigree, the ‘Xiangfei’ and ‘Zaomeiguixiang’ varieties are released by us, whereas the rest of the varieties were originated from foreign countries. In addition, the ‘Tamina’, ‘Muscat of Alexandria’, ‘Xiangfei’, ‘Italia’, and ‘Zaomeiguixiang’ varieties showed ‘muscat’ or ‘floral flavor’, whereas the ‘Christmas Rose’ and ‘Moldova’ varieties were classified as the neutral varieties. These varieties have different phenological periods. ‘Xiangfei’ and ‘Zaomeiguixiang’ were the early ripening type, the other five varieties belong to the middle to late ripening type. Each variety was sampled from veraison to harvest in four stages: the early stage of veraison (E-L 35), mid-maturity (E-L 36), end of veraison (E-L 37), and harvest (E-L 38). About 300 grapes were collected randomly from three vines in each variety at each developmental stage. During each sampling interval, the physicochemical indexes of each species were immediately determined (Figure S3). The remaining portion of the berries was immediately frozen in liquid nitrogen. Preserve frozen samples at −8 °C, until further analysis.

### 3.3. Extraction of Volatiles

Volatile compounds from grape berries were extracted according to the method we previously published, with minor modifications [31]. After dropping the pedicel and seeds, the grape berries (about 100 g) were ground then under liquid nitrogen mixed with 1 g of PVPP (polyvinyl poly-pyrrolidone). The resulting mixture was immersed for 4 h at 4 °C, then collected the clear liquid by centrifugation at 8000 rpm for 15 min at 4 °C. Afterwards, 5 mL of the liquid obtained above and 10 µL of the internal standard (1.00808 g/L) were added to a 15 mL vial, which contained 1 g of NaCl. Headspace solid-phase microextraction (HS-SPME) and Agilent 7890–5977 gas chromatography-mass spectrometer (GC-MS) were used for the absorption and analysis of volatile compounds. Each sample was subjected to two technical replicates.

### 3.4. Identification and Quantitation of Volatiles

Calculation of retention indices (RIs) of each component based on n-alkanes analyzed under the same chromatographic conditions. The volatile compounds were identified by comparing their RIs and mass spectrum with their standards and NIST11 library. The volatile compounds were quantified according to the previously published methods [32,33]. The synthetic grape berry juice matrix was prepared with tartaric acid and glucose according to the average sugar content (200 g/L) and acidity (7 g/L) in these berries. The pH of the matrix was then adjusted to 3.3. Mix all the monoterpenoid standards prepared in advance with the synthetic matrix to form the standard solution. The resultant solutions were sequentially diluted into 15 levels. The standard solutions were extracted and analyzed by the same method as the grape samples. The monoterpenoids in grape berries were quantified using their corresponding external standards. The compounds were quantified by compounds with the same number of C atoms and similar structures when no standards were available.

### 3.5. Extraction of Total RNA and Analysis by Real-Time qPCR

Total RNA extraction and transcript analysis of the key genes of the MEP pathway according to a method that has been published [34]. A total of 10 grape berries of each cultivar were removed from seeds and pedicels and then ground into powder. Total RNA was extracted from 100 mg of grape powder using the Total Plant RNA kit (Gene-Better Life Science, Beijing, China). The total RNA extraction for each grape variety was performed in duplicate. Afterwards, agarose gel electrophoresis and absorbance ratio (A260/A280, 1.8–2.0) on an Implen P330 nanophotometer (Implen GmbH, Munich, Germany) were used to verify the quality of RNA and to determine the concentration of RNA. cDNA was obtained from the qualified RNA through Reverse Transcription System Kit (Promega, Madison, WI, USA). The relative expression of key genes of the MEP pathway in five varieties was quantified by Real-time qPCR on the CFX Connect Real-Time System (Bio-Rad Laboratories, Hercules, CA, USA) using the SYBR green method. The real-time PCR system was programmed according to Martin [28]. The specific primers that were used in this study are listed in Table S1. *EF1-α*, *UBQ-L40*, and *Actin* with the GenBank accession number EC959059, EC929411, and EC969944, respectively, were used as internal controls according to previous research [35]. Calculation of normalized expression of the target genes by the difference between the cycle threshold (Ct) of target and reference genes (∆CT = Ct Target-Ct Ref Gene) [36].

### 3.6. Genotyping of the snp1822

The genomic DNA was extracted from the young leaves of the seven varieties by CTAB methods [37,38]. The following primers were designed to amplify the full-ORF *VvDXS* cDNA(2151 bp) containing the 1822 sites, forward primer: CTGTGGACATTCCATCAATTTG and reverse primer: AATGGCAACAAGGTCATCTATG, the PCR conditions were based on Battilana’s research [39]. The amplified PCR products were recovered by recovery kits (DP209, Tiangen, Beijing, China) and then sanger sequenced by Sangon Biotech.

### 3.7. Statistical Analysis

SPSS 20.0 (SPSS Inc., Chicago, IL, USA) was used to take the One-way and two-factor ANOVA analysis and Pearson’s correlation analysis. The one-way ANOVA analysis was performed using R packages ‘agricolae’ in R (3.6.1). The heat plots were generated by the ‘pheatmap’ package in R (3.6.1). Principal component analysis (PCA) was conducted on MetaboAnalyst 5.0 (https://www.metaboanalyst.ca/MetaboAnalyst/home.xhtml, accessed on 16 May 2022) using Auto scaling in normalization procedure. Drawing line and bar charts with Origin 8.0 (OriginLab., Northampton, MA, USA).

## 4. Conclusions

In conclusion, the ‘Xiangfei’ variety was found to possess the highest concentrations of monoterpenoids at harvest, while the ripe ‘Moldova’ variety had the lowest levels of total monoterpenoids in both the 2013 and 2014 seasons. Linalool, geraniol, *β*-Citronellol, *trans*-furan linalool oxide, and *cis*-furan linalool oxide seem to make important contributions to the overall aroma of these table grape varieties. Regarding their evolution patterns in berry development, these table grapes were divided into three clusters, and the expression pattern of MEP pathway related genes could regulate their accumulation. Our study found that the genotype of 1822snp of *DXS1* in table grapes could explain to some extent the differences in monoterpenoids content among varieties. Meanwhile, our results revealed that there was an important role of *VvDXS3* gene expression in the regulation of monoterpenoid accumulation in these table grape varieties. This study enhanced our knowledge of the differences in monoterpenoids accumulation in table grapes and their regulation mechanisms.

## Figures and Tables

**Table 1:** Concentrations of monoterpenoids compounds in different table grape varieties at harvest in 2013 (μg/L). “nd” represented “Not detected”. “tr” represented “Trace amount”. Data were shown as means ± standard deviations of duplicate tests. Values with different letters (a–f) on a row were significantly different based on Tukey’s multiple range test (*p* < 0.05). ‘Alexandria’ represented ‘Muscat of Alexandria’, ‘Zmgx’ represented ‘Zaomeiguixiang’.

Compounds	Moldova	Christmas Rose	Alexandria	Italia	Tamina	Xiangfei	Zmgx
*β*-Myrcene	tr	0.09 ± 0.01 d	0.86 ± 0.01 c	0.97 ± 0.01 c	1.13 ± 0.04 c	2.08 ± 0.59 b	3.59 ± 0.22 a
Limonene	0.10 ± 0.03 d	0.30 ± 0.18 d	0.33 ± 0.00 d	0.94 ± 0.00 cd	1.21 ± 0.06 bc	2.75 ± 0.97 a	2.06 ± 0.04 ab
*β*-*trans*-Ocimene	nd	nd	0.21 ± 0.00 c	0.31 ± 0.07 c	0.73 ± 0.26 b	1.29 ± 0.34 a	1.51 ± 0.07 a
*β*-*cis*-Ocimene	nd	0.05 ± 0.01 c	0.52 ± 0.01 bc	0.94 ± 0.00 b	1.07 ± 0.03 b	2.14 ± 0.82 a	2.63 ± 0.19 a
*γ*-Terpinen	tr	nd	0.04 ± 0.00 b	0.07 ± 0.00 b	0.15 ± 0.01 b	0.42 ± 0.21 a	0.41 ± 0.07 a
Terpinolene	0.02 ± 0.00 f	nd	0.16 ± 0.01 e	0.24 ± 0.01 d	0.47 ± 0.03 c	1.86 ± 0.06 a	1.74 ± 0.01 b
*trans*-Rose oxide	nd	nd	0.05 ± 0.00 c	0.05 ± 0.00 c	0.06 ± 0.00 bc	0.21 ± 0.17 b	0.36 ± 0.02 a
*cis*-Rose oxide	nd	nd	0.06 ± 0.00 c	0.06 ± 0.00 c	0.08 ± 0.00 c	0.59 ± 0.54 b	1.26 ± 0.04 a
(*trans*,*cis*)-Allo-ocimene	0.02 ± 0.00 d	nd	0.35 ± 0.01 c	0.39 ± 0.08 c	0.94 ± 0.07 b	2.53 ± 0.29 a	2.42 ± 0.04 a
Allo-ocimene	0.02 ± 0.00 c	nd	0.24 ± 0.01 c	0.21 ± 0.06 c	0.84 ± 0.06 b	2.03 ± 0.29 a	1.78 ± 0.05 a
*trans*-furan linalool oxide	2.69 ± 0.01 b	1.44 ± 2.03 b	4.01 ± 1.43 b	5.19 ± 0.32 b	6.64 ± 0.87 b	283.97 ± 17.99 a	17.96 ± 3.47 b
*cis*-Furan linalool oxide	2.72 ± 0.01 c	1.62 ± 2.29 c	3.44 ± 0.01 c	9.79 ± 0.73 c	8.99 ± 1.36 c	315.83 ± 23.07 a	50.62 ± 9.76 b
Citronellal	1.34 ± 1.89 de	nd	3.85 ± 0.00 bc	2.97 ± 0.12 cd	7.46 ± 0.69 a	5.43 ± 0.98 ab	6.19 ± 0.93 a
Nerol oxide	1.35 ± 1.90 c	3.01 ± 0.18 c	3.83 ± 1.19 c	3.31 ± 0.71 c	4.87 ± 0.80 c	47.62 ± 3.22 a	25.85 ± 8.85 b
Linalool	0.14 ± 0.00 c	0.14 ± 0.01 c	7.17 ± 0.01 c	11.87 ± 0.88 c	16.39 ± 0.13 bc	46.93 ± 24.60 a	35.38 ± 2.49 ab
4-Terpineol	tr	nd	0.05 ± 0.00 d	0.06 ± 0.01 c	0.10 ± 0.00 b	0.31 ± 0.01 a	0.09 ± 0.01 b
Neral	0.02 ± 0.00 d	nd	0.19 ± 0.00 cd	0.46 ± 0.06 b	0.32 ± 0.01 bc	0.29 ± 0.10 bc	0.73 ± 0.25 a
*α*-Terpineol	0.07 ± 0.00 d	0.03 ± 0.03 d	0.89 ± 0.00 cd	1.41 ± 0.25 c	5.54 ± 0.18 b	10.93 ± 1.34 a	5.15 ± 0.11 b
Geranial	0.15 ± 0.00 c	0.16 ± 0.02 c	1.28 ± 0.02 bc	2.56 ± 0.33 b	2.18 ± 0.12 bc	1.51 ± 0.31 bc	4.97 ± 2.30 a
*β*-Citronellol	1.19 ± 0.04 cd	nd	4.08 ± 0.02 cd	4.17 ± 0.10 cd	4.87 ± 0.10 c	10.47 ± 3.96 b	20.98 ± 2.79 a
*γ*-Geraniol	0.99 ± 0.04 d	0.89 ± 0.13 d	3.31 ± 0.01 c	3.43 ± 0.12 bc	4.76 ± 0.25 b	4.09 ± 0.80 bc	10.36 ± 1.32 a
Nerol	tr	tr	0.17 ± 0.00 b	0.17 ± 0.00 b	0.20 ± 0.01 b	0.22 ± 0.03 b	0.47 ± 0.06 a
Isogeraniol	tr	nd	nd	tr	tr	nd	tr
Geraniol	1.48 ± 0.07 d	2.30 ± 0.31 d	25.10 ± 0.27 c	18.34 ± 0.88 c	54.76 ± 4.44 b	26.37 ± 3.35 c	74.75 ± 9.71 a
Geranic acid	tr	0.42 ± 0.07 c	5.73 ± 0.14 b	5.68 ± 3.33 b	15.19 ± 0.96 a	15.95 ± 0.16 a	16.44 ± 1.64 a

**Table 2:** Concentrations of monoterpenoids compounds in different table grape varieties at harvest in 2014 (μg/L). “nd” represented “Not detected”. “tr” represented “Trace amount”. Data were shown as means ± standard deviations of duplicate tests. Values with different letters (a–f) on a row were significantly different based on Tukey’s multiple range test (*p* < 0.05). ‘Alexandria’ represented ‘Muscat of Alexandria’, ‘Zmgx’ represented ‘Zaomeiguixiang’.

Compounds	Moldova	Christmas Rose	Alexandria	Italia	Tamina	Xiangfei	Zmgx
*β*-Myrcene	0.01 ± 0.01 e	0.09 ± 0.00 e	2.00 ± 0.02 d	5.29 ± 0.03 b	4.98 ± 0.16 b	17.18 ± 0.32 a	4.45 ± 0.03 c
Limonene	nd	nd	1.35 ± 0.00 e	3.48 ± 0.01 b	3.26 ± 0.01 c	14.94 ± 0.10 a	3.07 ± 0.03 d
*β*-*trans*-Ocimene	0.02 ± 0.01 e	nd	1.46 ± 0.00 d	3.35 ± 0.00 b	3.21 ± 0.01 b	11.04 ± 0.20 a	2.29 ± 0.01 c
*β*-*cis*-Ocimene	nd	nd	1.68 ± 0.01 d	4.83 ± 0.00 b	4.80 ± 0.21 b	17.77 ± 0.33 a	3.21 ± 0.00 c
*γ*-Terpinen	nd	nd	0.93 ± 0.01 a	1.36 ± 0.02 a	3.67 ± 3.31 a	3.38 ± 0.05 a	1.17 ± 0.00 a
Terpinolene	nd	nd	2.48 ± 0.00 c	3.27 ± 0.04 b	3.19 ± 0.16 b	8.49 ± 0.03 a	2.44 ± 0.02 c
*trans*-Rose oxide	nd	nd	0.10 ± 0.00 d	0.25 ± 0.01 c	nd	0.80 ± 0.05 b	1.80 ± 0.01 a
*cis*-Rose oxide	nd	nd	0.51 ± 0.02 d	0.95 ± 0.01 c	0.26 ± 0.02 e	1.93 ± 0.04 b	2.30 ± 0.03 a
(*trans*,*cis*)-Allo-ocimene	nd	nd	0.27 ± 0.00 d	1.42 ± 0.01 b	1.40 ± 0.01 b	2.73 ± 0.00 a	1.32 ± 0.00 c
Allo-Ocimene	nd	nd	1.64 ± 0.00 e	4.62 ± 0.08 b	4.34 ± 0.16 c	22.12 ± 0.23 a	2.38 ± 0.03 d
*trans*-furan linalool oxide	nd	nd	4.77 ± 0.01 d	12.19 ± 0.39 b	9.48 ± 0.22 c	207.16 ± 1.16 a	4.08 ± 0.19 d
*cis*-furan linalool oxide	nd	nd	11.72 ± 0.01 e	59.05 ± 1.35 b	15.41 ± 0.01 c	227.66 ± 0.82 a	13.31 ± 0.13 d
Citronellal	nd	nd	nd	nd	4.70 ± 0.66 b	13.57 ± 0.14 a	5.24 ± 0.02 b
nerol oxide	nd	nd	41.87 ± 0.16 b	22.94 ± 0.76 d	24.16 ± 0.57 d	201.86 ± 4.84 a	35.04 ± 2.09 c
Linalool	0.16 ± 0.00 e	0.14 ± 0.01 e	9.14 ± 0.06 d	74.85 ± 0.16 b	61.33 ± 1.36 c	329.59 ± 7.56 a	14.32 ± 0.26 d
4-Terpineol	nd	nd	0.13 ± 0.00 d	0.21 ± 0.00 c	0.27 ± 0.00 b	1.06 ± 0.02 a	0.13 ± 0.01 d
Neral	nd	nd	nd	0.20 ± 0.00 b	0.23 ± 0.04 b	0.23 ± 0.01 b	0.51 ± 0.00 a
*α*-Terpineol	0.02 ± 0.03 d	nd	6.68 ± 0.00 c	8.61 ± 0.21 b	9.29 ± 0.14 b	26.02 ± 0.82 a	6.21 ± 0.06 c
Geranial	nd	nd	0.77 ± 0.00 d	1.00 ± 0.00 cd	1.55 ± 0.38 b	1.28 ± 0.09 bc	2.53 ± 0.03 a
*β*-Citronellol	nd	nd	4.53 ± 0.00 d	5.99 ± 0.04 c	4.64 ± 0.10 d	10.00 ± 0.13 b	43.62 ± 1.41 a
*γ*-geraniol	nd	nd	7.48 ± 0.01 c	7.99 ± 0.02 b	7.91 ± 0.05 b	7.87 ± 0.05 b	12.29 ± 0.13 a
Nerol	tr	tr	0.06 ± 0.00 d	0.22 ± 0.00 c	0.21 ± 0.00 c	0.30 ± 0.00 b	0.93 ± 0.03 a
Isogeraniol	nd	nd	0.10 ± 0.00 d	0.11 ± 0.00 c	0.12 ± 0.01 c	0.13 ± 0.00 b	0.27 ± 0.01 a
Geraniol	0.41 ± 0.00 f	1.62 ± 0.05 f	16.40 ± 0.14 e	26.35 ± 0.24 d	37.46 ± 0.80 c	48.19 ± 0.34 b	102.37 ± 3.06 a
Geranic acid	0.14 ± 0.19 e	nd	13.20 ± 0.41 d	60.30 ± 7.50 b	6.61 ± 0.93 de	41.08 ± 5.74 c	84.75 ± 2.84 a

**Table 3:** Two-way ANOVA analysis of monoterpenoid concentrations in seven table grape varieties during the development of berries.

Compounds	Variety			Season			Variety × Season		
	F Value	*p*	Sig.	F Value	*p*	Sig.	F Value	*p*	Sig.
*β*-Myrcene	12.99	9.30 × 10^−^^11^	***	34.67	5.48 × 10^−^^8^	***	5.09	1.38 × 10^−^^4^	***
Limonene	14.81	5.52 × 10^−^^12^	***	22.44	7.00 × 10^−^^6^	***	4.81	2.41 × 10^−^^4^	***
*β*-*trans*-Ocimene	10.81	3.31 × 10^−^^9^	***	43.93	1.86 × 10^−^^9^	***	6.11	1.80 × 10^−^^5^	***
*β*-*cis*-Ocimene	8.85	1.04 × 10^−^^7^	***	25.30	2.00 × 10^−^^6^	***	4.20	8.39 × 10^−^^4^	***
*γ*-Terpinen	2.03	6.83 × 10^−^^2^		13.81	3.36 × 10^−^^4^	***	1.67	1.36 × 10^−^^1^	
Terpinolen	24.46	1.57 × 10^−^^17^	***	104.28	4.21 × 10^−^^17^	***	8.80	1.13 × 10^−^^7^	***
*trans*-Rose oxide	7.90	5.87 × 10^−^^7^	***	12.57	6.03 × 10^−^^4^	***	3.99	1.30 × 10^−^^3^	**
*cis*-Rose oxide	31.77	5.88 × 10^−^^21^	***	33.93	7.27 × 10^−^^8^	***	5.62	4.77 × 10^−^^5^	***
(*trans*,*cis*)-Allo-ocimene	29.67	4.99 × 10^−^^20^	***	0.33	5.67 × 10^−^^1^		4.37	5.90 × 10^−^^4^	***
Allo-ocimene	12.25	3.04 × 10^−^^10^	***	30.52	2.72 × 10^−^^7^	***	6.67	6.12 × 10^−^^6^	***
*trans*-furan linalool oxide	37.79	2.03 × 10^−^^23^	***	1.39	2.41 × 10^−^^1^		3.36	4.67 × 10^−^^3^	**
*cis*-furan linalool oxide	50.36	8.49 × 10^−^^28^	***	6.85	1.03 × 10^−^^2^		7.64	9.66 × 10^−^^7^	***
Citronellal	1.00	4.31 × 10^−^^1^		0.99	3.22 × 10^−^^1^		1.00	4.30 × 10^−^^1^	
Nerol oxide	17.42	1.27 × 10^−^^13^	***	16.78	8.65 × 10^−^^5^	***	4.04	1.15 × 10^−^^3^	**
Linalool	12.68	1.53 × 10^−^^10^	***	12.49	6.27 × 10^−^^4^	***	5.26	9.76 × 10^−^^5^	***
4-Terpineol	41.38	9.18 × 10^−^^25^	***	25.31	2.21 × 10^−^^6^	***	5.20	1.12 × 10^−^^4^	***
Neral	24.33	1.81 × 10^−^^17^	***	7.26	8.31 × 10^−^^3^	**	0.82	5.55 × 10^−^^1^	
*α*-Terpineol	28.37	1.98 × 10^−^^19^	***	29.30	4.42 × 10^−^^7^	***	2.92	1.16 × 10^−^^2^	
Geranial	19.35	9.12 × 10^−^^15^	***	7.09	9.04 × 10^−^^3^	**	0.99	4.38 × 10^−^^1^	
*β*-Citronellol	22.74	1.23 × 10^−^^16^	***	4.44	3.77 × 10^−^^2^		5.08	1.42 × 10^−^^4^	***
*γ*-Geraniol	4.04	1.16 × 10^−^^3^	**	7.51	7.30 × 10^−^^3^	**	1.47	1.97 × 10^−^^1^	
Nerol	22.19	2.40 × 10^−^^16^	***	3.64	5.90 × 10^−^^2^		1.98	7.50 × 10^−^^2^	
Isogeraniol	12.74	1.39 × 10^−^^10^	***	394.71	3.85 × 10^−36^	***	11.50	1.05 × 10^−^^9^	***
Geraniol	28.95	1.06 × 10^−^^19^	***	1.01	3.19 × 10^−^^1^		0.94	4.72 × 10^−^^1^	
Geranic acid	22.40	1.84 × 10^−^^16^	***	30.37	2.88 × 10^−^^7^	***	12.11	3.80 × 10^−^^10^	***

F values of the variety, Vintage and their interactions were evaluated using monoterpenoid concentrations in table grapes at different developmental stages. ** and *** indicated the significance effects at *p* < 0.01 and 0.001.

**Table 4:** Pearson’s correlation between terpene volatiles and MEP pathway gene expression in seven table grape varieties.

	*VvDXS1*	*VvDXS3*	*VvDXR*	*VvHDR*	*VvGPPS*
*β*-Myrcene	0.482 *	0.609 **	0.410	0.351	0.426
Limonene	0.737 **	0.652 **	0.511 *	0.391	0.576 **
*β*-*trans*-Ocimene	0.643 **	0.728 **	0.498 *	0.457 *	0.534 *
*β*-*cis*-Ocimene	0.599 **	0.668 **	0.430	0.360	0.473 *
*γ*-Terpinen	0.661 **	0.651 **	0.441	0.314	0.505 *
Terpinolen	0.587 **	0.556 *	0.346	0.232	0.443
*trans*-Rose oxide	0.467 *	0.573 **	0.336	0.244	0.390
*cis*-Rose oxide	0.411	0.518 *	0.270	0.170	0.316
(*trans*,*cis*)-Allo-ocimene	0.636 **	0.598 **	0.427	0.326	0.455 *
Allo-Ocimene	0.682 **	0.644 **	0.408	0.379	0.509 *
*trans*-furan linalool oxide	0.713 **	0.323	0.223	−0.006	0.370
*cis*-furan linalool oxide	0.684 **	0.365	0.264	0.029	0.378
Citronellal	0.550 *	0.749 **	0.715 **	0.786 **	0.733 **
Nerol oxide	0.518 *	0.426	0.150	0.004	0.309
Linalool	0.693 **	0.598 **	0.441	0.301	0.503 *
4-Terpineol	0.666 **	0.434	0.338	0.132	0.432
Neral	0.359	0.626 **	0.472 *	0.489*	0.447 *
*α*-Terpineol	0.676 **	0.619 **	0.449 *	0.330	0.506 *
Geranial	0.252	0.589 **	0.449 *	0.490 *	0.402
*β*-Citronellol	0.366	0.528 *	0.264	0.215	0.300
*γ*-Geraniol	0.329	0.609 **	0.510 *	0.533 *	0.494 *
Geraniol	0.392	0.648 **	0.424	0.449 *	0.429
Nerol	0.397	0.686 **	0.774 **	0.819 **	0.716 **
Isogeraniol	0.334	0.693 **	0.599 **	0.666 **	0.540 *
Geranic acid	0.543 *	0.715 **	0.425	0.434	0.435

* and ** indicate the significance effect at *p* < 0.05 and 0.01.

## Data Availability

Not applicable.

## Supplementary Materials

The following supporting information can be downloaded at: https://www.mdpi.com/article/10.3390/plants11162143/s1, Figure S1: Sequencing peak map of the genotype snp1822 in *DXS1* of seven varieties; Figure S2: Photographs of seven table grapes at harvest and their genealogical relationships; Figure S3: Total soluble solids (TSS) and pH of seven table grape varieties during berry development in 2013 and 2014. Table S1: Real-time qPCR primers used in the study.
