# Supplementary material for: Monoterpenoids Evolution and MEP Pathway Gene Expression Profiles in Seven Table Grape Varieties
Source: Plants (Basel). 2022 Aug 18;11(16):2143. doi: 10.3390/plants11162143 (PMC9413098; doi:10.3390/plants11162143)
Supplement: Supplementary file 1 [file plants-11-02143-s001.zip › plants-1782589-supplementary.pdf]

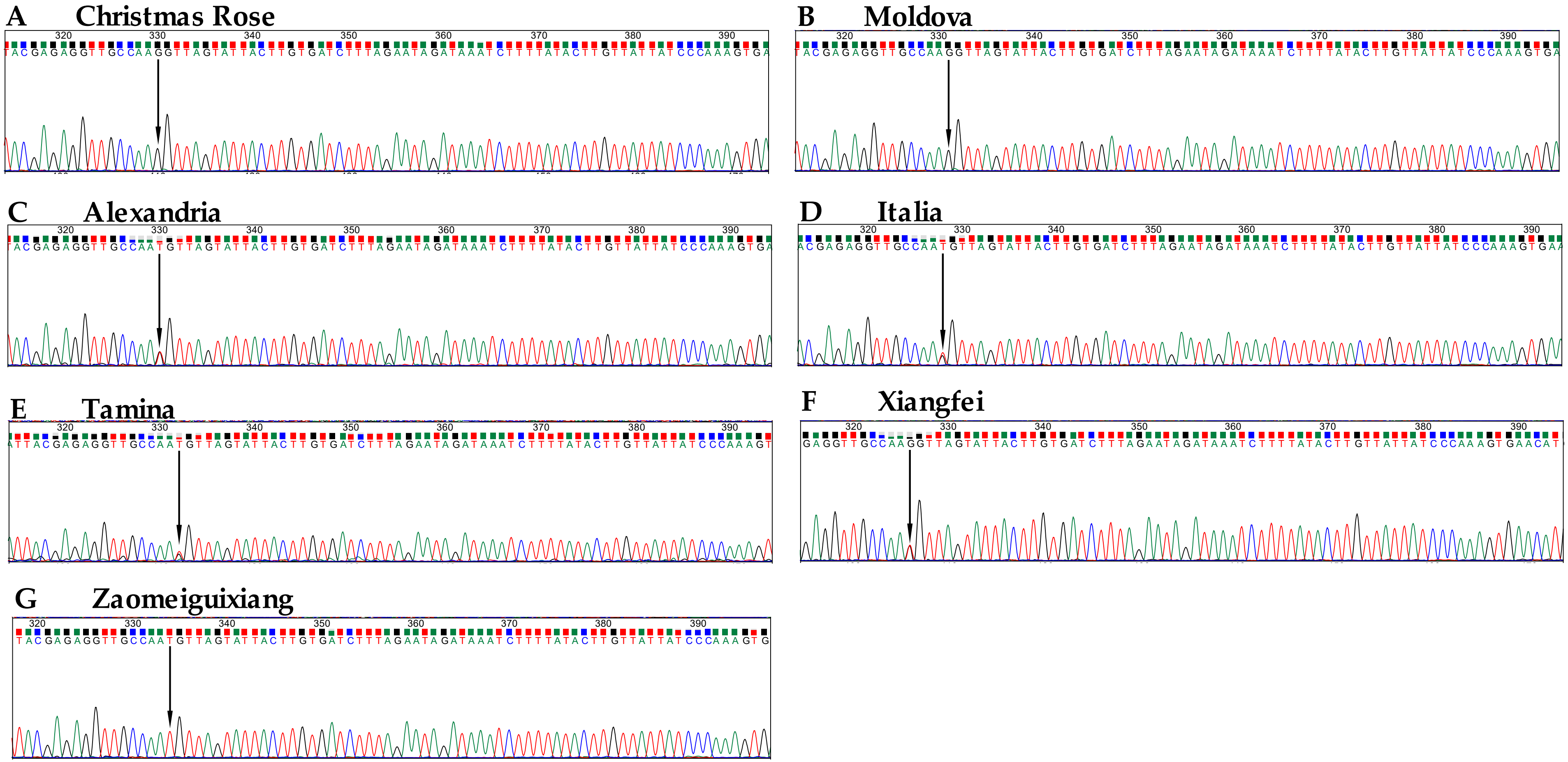

Figure S1. Sequencing peak map of the genotype snp1822 in *DXS1* of seven varieties. (A) Christmas Rose; (B) Moldova; (C) Muscat of Alexandria; (D) Italia; (E) Tamina; (F) Xiangfei; (G) Zaomeiguixiang.

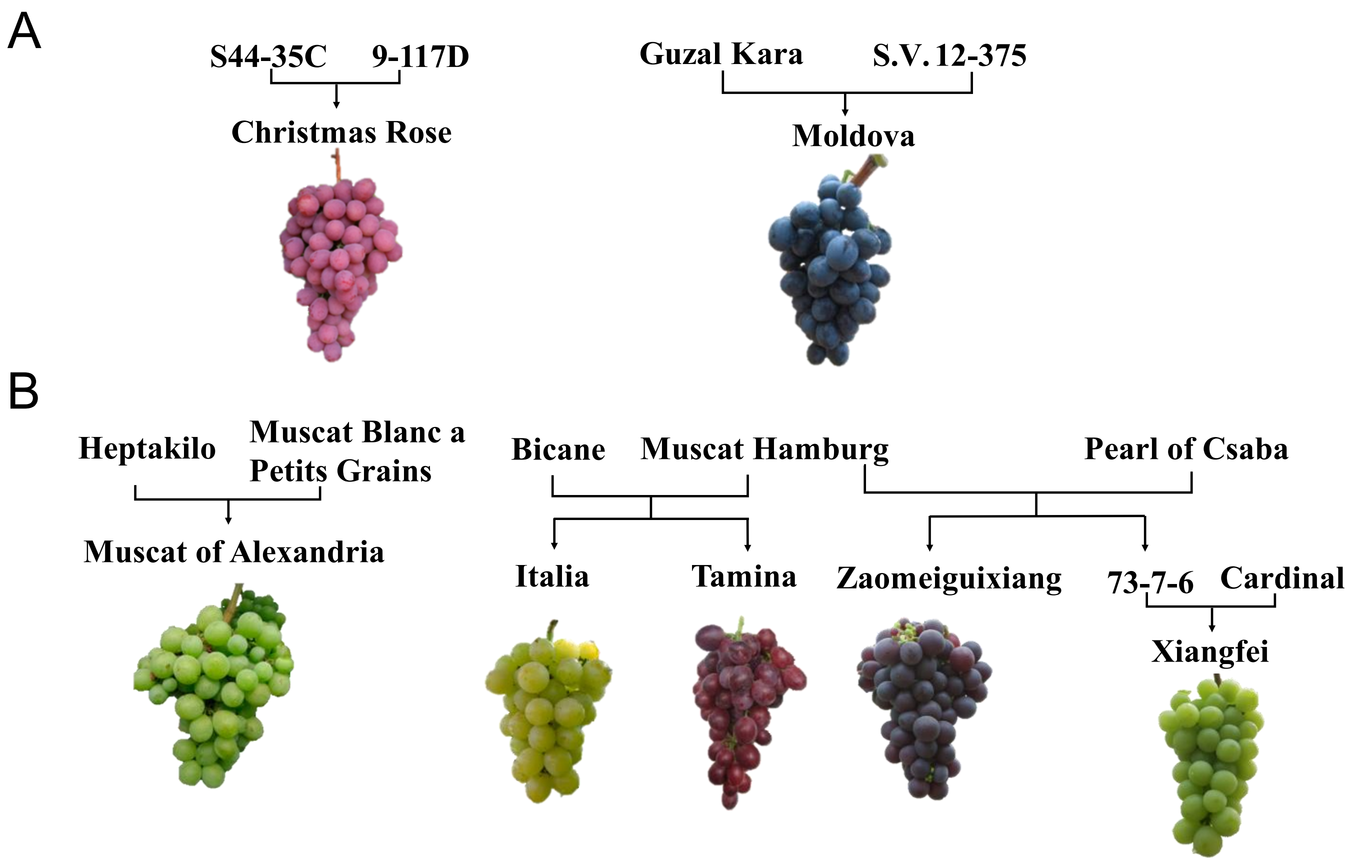

Figure S2. Photographs of seven table grapes at harvest and their genealogical relationships. (A) Two non-muscat table grapes 'Chrismas Rose' and 'Moldova'; (B) five muscat table grapes 'Muscat of Alexandria', 'Italia', 'Tamina', Zaomeiguixiang' and 'Xiangfei'.

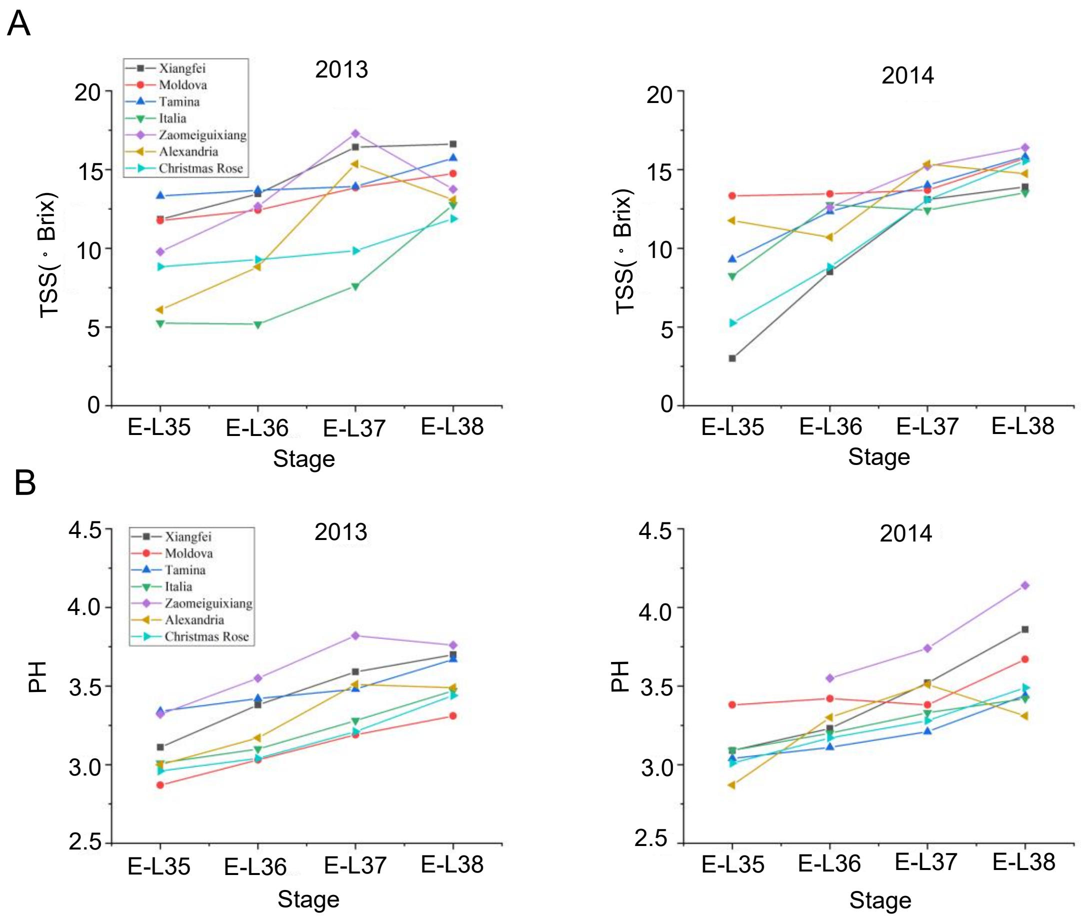

Figure S3. Total soluble solids (TSS) and pH of seven table grape cultivars during berry development in 2013 and 2014. (A) Total soluble solids (TSS) of seven table grapes in two years; (B) PH of seven table grapes in two years. 'Alexandria' represented 'Muscat of Alexandria'.

Table S1. Real-time qPCR primers used in the study.

Gene name	Forward primer	Reverse primer
VvDXS1	CTCATTTCCTGCCCATTTTAGC	CTTACTCCTTTGCTGGGATTGG
VvDXS3	GAAGGCTCTGTTGGAGGGTTT	TCCTCTGGTGATGCCTGTTCT
VvDXR	AGAGGCTTTGGCTGACTGTGA	AACCTGCGCAACCTACTATTCC
VvHDR	TCTTCCTCGTCTGTGGCTGTT	GCGATTCATGAGCTCCAGAG
VvGPPS	AGAATCTGGGATTGGCATTCC	TGGCGGATGTCAGACAATGA
EF1-α	GTGCGTCATAGTTTTCTGCCTTCTTCCTTG	CTCAACCAGTTATCTGCCACCGCCTATC
UBQ-L40	CATAACATTTGCGGCAGATCA	TGGTGGTATTATTGAGCCATCCTT
Actin	GCATCCCTCAGCACCTTCCAGCAG	CCACCTCAACACATCTCCATGTCAACC
